# Multidetector Computed Tomographic Angiography for Optimal Cartography of the Visceral Abdominal Arterial Network: An Extensive Pictorial Review with Emphasis on Common and Uncommon Collateral Pathways, Complications and some Specific Syndromes

**DOI:** 10.5334/jbr-btr.1203

**Published:** 2017-02-01

**Authors:** Bruno Coulier

**Affiliations:** 1 Clinique Saint-Luc, Bouge, Belgium, BE

**Keywords:** Multidetector Computed Tomographic Angiography, Collateral pathway, Collateral Circulation, Compensary bloodflow, Arc of Riolan, Meandering Artery, Median Arcuate Ligament Syndrome, Leriche’s Syndrome, Aorto-iliac occlusive disease, Chronic Mesenteric Ischemia, Aneurysm, Mesenteric Arteries, Celiac Trunk

## Abstract

Multidetector computed tomographic angiography (MDCTA) is the new gold standard for diagnostic evaluation of the abdominal and/or mesenteric arteries. It is not invasive and provides a 2D and 3D global cartography of all abdominal arteries and that with only a limited amount of contrast media.

MDCTA allows the optimal diagnosis of single or multiple arterial stenosis and easily analyses sometimes very complex collateral pathways. It constitutes a major advance to plan the arterial visceral safety of major commonly performed abdominal surgical procedures such as aorto-iliac surgery, endovascular aneurysm repair (EVAR), but also complex pancreatic and gastrointestinal or colonic surgery. It also allows to plan the most optimal strategy for revascularization of the mesenteric system through percutaneous angioplasty, stent placement or surgical bypass.

This extensive pictorial review illustrates a large variety of situations which may be found during clinical practise. Single compression or stenosis of each digestive artery, combined and/or complex associations of stenosis and/or compressions of several arteries, secondary complications like aneurysms and classical but also sometimes unusual patterns of collateralization are richly illustrated. Specific syndromes comprising the median arcuate ligament syndrome (MALS) and the Leriche’s syndrome are also discussed.

## Introduction

Today the panel of imaging techniques available to investigate abdominal vessels is varied comprising of duplex and color flow Doppler sonography, angio-CT, angio-MRI and conventional angiography (CA). The choice of one technique over another depends on its availability, the clinical circumstances, the degree of emergency but also on the patient’s physical characteristics, age and renal function.

Multidetector computed tomographic angiography (MDCTA) today represents the best choice when the purpose of the examination is to quickly and easily obtain optimal global and non-invasive analysis and/or cartography of the abdominal and visceral vessels with all their interconnections. In this context MDCTA represents the new gold standard and outperforms CA. MDCTA has major advantages on CA. First, it is a very fast and non-invasive technique which only requires a limited amount of intravenous contrast media to provide high quality 2D and 3D anatomic images. Then a global cartography of all arteries may be obtained simultaneously, an opportunity that cannot be meet during CA. Multiple successive selective or semi-selective invasive catheterizations would be necessary to obtain such a global analysis of all abdominal vessels during CA. MDCTA has only several limitations. The detection threshold of the tiny arteries is lower than with CA and the opacification is static when compared to the dynamic aspect of CA which can better detect the direction of blood flow particularly in the collateral pathways. The fact that all arteries are opacified simultaneously can make spatial analysis difficult. Fortunately, secondary high quality selective reconstructions are able to dissociate and analyse the complexity of arterial superposition anatomic overlays. This nevertheless requires a good skill in the use of 3D post-processing programs.

MDCTA offers the opportunity to quickly diagnose or rule out mesenteric stenosis or compression in patients presenting with suggestive abdominal pain or angina. It also constitutes a primordial advance progress to plan the arterial safety of many major abdominal surgical procedures comprising classical aorto-iliac surgery, endovascular aneurysm repair (EVAR), complex pancreatic and gastrointestinal or colonic surgery but also to optimally plan revascularization of the mesenteric system through percutaneous angioplasty (PTA), stent placement or surgical bypass [1].

During embryogenesis, most segmental arteries regress and only three dominant major mesenteric visceral arteries persist: the celiac trunk (CTK), the superior mesenteric artery (SMA) and the inferior mesenteric artery (IMA) [1]. Fortunately, this mesenteric circulation has or may develop an extensive collateral network to ensure sufficient blood supply and in most cases these interconnections may easily supply if significant stenosis develops in one major artery.

Previous studies have suggested that a significant stenosis of at least two of the three main digestive arteries must occur and/or that a complete occlusion of the CTK must precede the occlusion of the rest of the mesenteric arteries before the occurrence of clinical symptoms of mesenteric angina [2]. Therefore, complaints related to symptomatic stenotic disease or chronic mesenteric ischemia which represents a serious and complex vascular disorder remains a rather rare event when compared with the high prevalence of chronic mesenteric atheromatous disease [1,3]. A stenosis of at least 70% in the mesenteric arteries may be considered as the cut-off for collateral development and increased compensatory blood flow [3].

This extensive pictorial review illustrates a large variety of situations which may be found during clinical practise. Single compression or stenosis of each digestive artery, combined and/or complex associations of stenosis and/or compressions of several arteries, secondary complications like aneurysms and classical but also sometimes unusual patterns of collateralizations are richly illustrated. Specific syndromes such as the median arcuate ligament syndrome (MALS) and the Leriche’s syndrome that are also discussed.

### Compression and stenosis of the celiac trunk (CTK)

The incidence of hemodynamically significant CTK stenosis in an asymptomatic population has been evaluated to 7.3% and the most important etiology is extrinsic compression by the median arcuate ligament (MAL) of the diaphragm. Atherosclerosis remains only a rather minor cause of stenosis of the CTK [4]. The MAL is a fibrous arch that connects the right and left diaphragmatic crura and defines the anterior margin of the aortic hiatus [5].

The Dunbar syndrome induced by the celiac trunk compression syndrome (CTCS) also called the median arcuate ligament syndrome (MALS) is a potential clinical entity characterized by a triad comprising epigastric pain, weight loss and postprandial pain with nausea and vomiting [5,6,7,8,9,10]. These symptoms are believed to be secondary to intermittent ischemia especially during the expiration phase [8]. These symptoms are attenuated when the patient is in an erect position and during inspiration [6,9]. Indeed, in these positions the CTK descends in the abdominal cavity and becomes more vertical resulting in a relief or attenuation of the compression [7,9,11,12].

Atypical manifestations of the MALS are extremely variable ranging from exercise related pain and diarrhea in elite athletes to dramatic rupture of a secondary pancreaticoduodenal artery aneurysm (PDAA) developing on collaterals [5,8]. Nevertheless, the hypothesis that CTCS may lead or not to the clinical picture of MALS remains controversial [6,13]. Indeed nearly 13 to 50% of asymptomatic patients may exhibit a variable degree of compression during imaging. Thus, not only the compression but also the symptoms must then be simultaneously present to allow the diagnosis of MALS.

The physiopathology of MALS is also controversial: are the symptoms really caused by ischemia of the gut itself or by neurogenic compression or ischemia of the celiac ganglion [9]?

**Figure 1:**
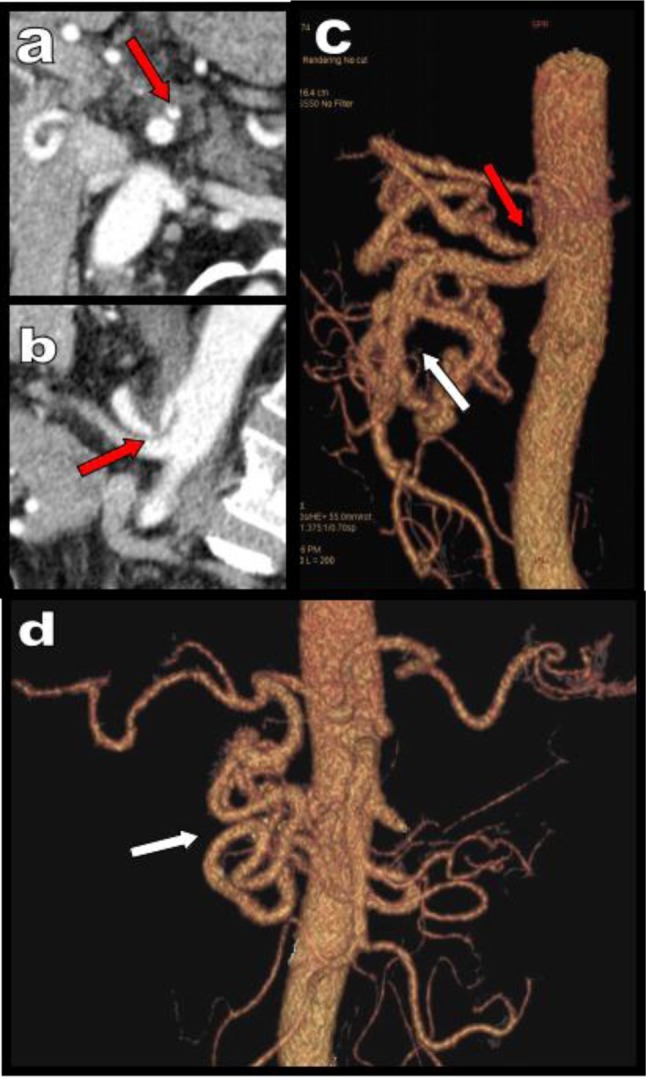
Axial view **(a)**, sagital MPR **(b)** and corresponding VR views **(c, d)** illustrate a typical case of high degree compression of the CTK (red arrows) by the MAL of the diaphragm fortuitously diagnosed in a 68-year-old patient. The compression appears well compensated by retrograde arterial supply from the SMA through typical serpiginous hypertrophy of the PDAs (white arrows).

**Figure 2:**
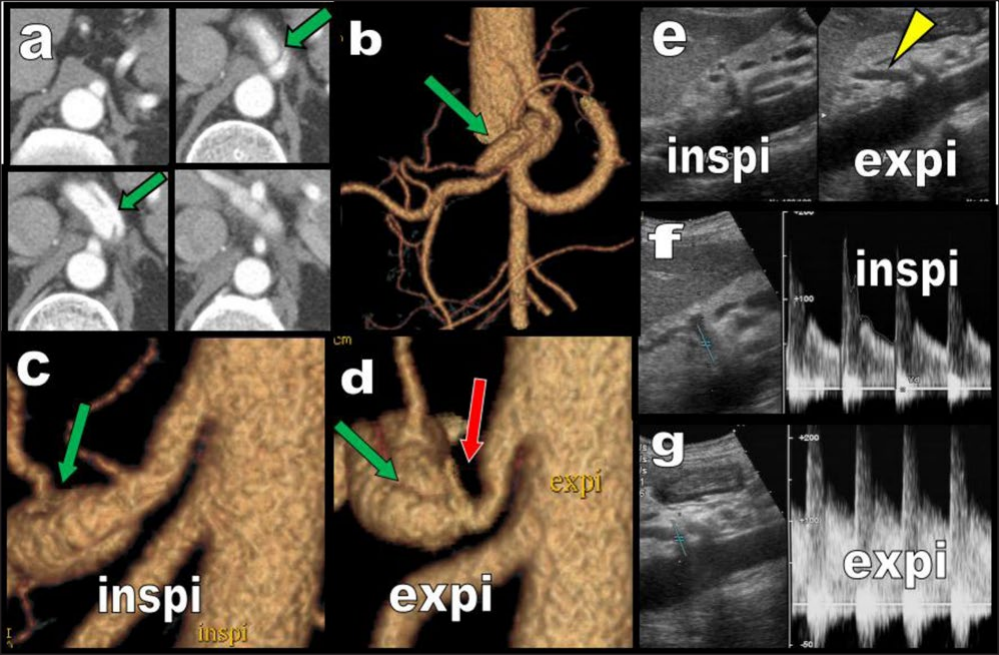
Axials **(a)** and VR views **(b, c, d)** obtained in a 50-year-old patient presenting with epigastric pain. Post ostial segmental ectasic dissection of the CTK is found (green arrows). The deformation of the proximal CTK during inspiration **(c)** is limited but compression by the MAL is clearly obvious on expiratory imaging **(d)** especially on VR views showing the typical deep notch caused by the MAL (red arrow). Continuous iterative compression of the CTK by the MAL can reasonably be proposed as an explanation for the arterial dissection. During Duplex Doppler Ultrasound **(e, f, g)** the compression and/or the deformation of the CTK by the MAL may be better demonstrated during expiration **(e, g)** as illustrated in this other case found in a young man. The CTK bends vertically during expiration (yellow arrowhead) and Doppler spectral images of the proximal CTK during expiration **(g)** show turbulences and accelerated peak systolic and end diastolic velocities when compared with Doppler spectral images obtained during inspiration **(f)**.

**Figure 3:**
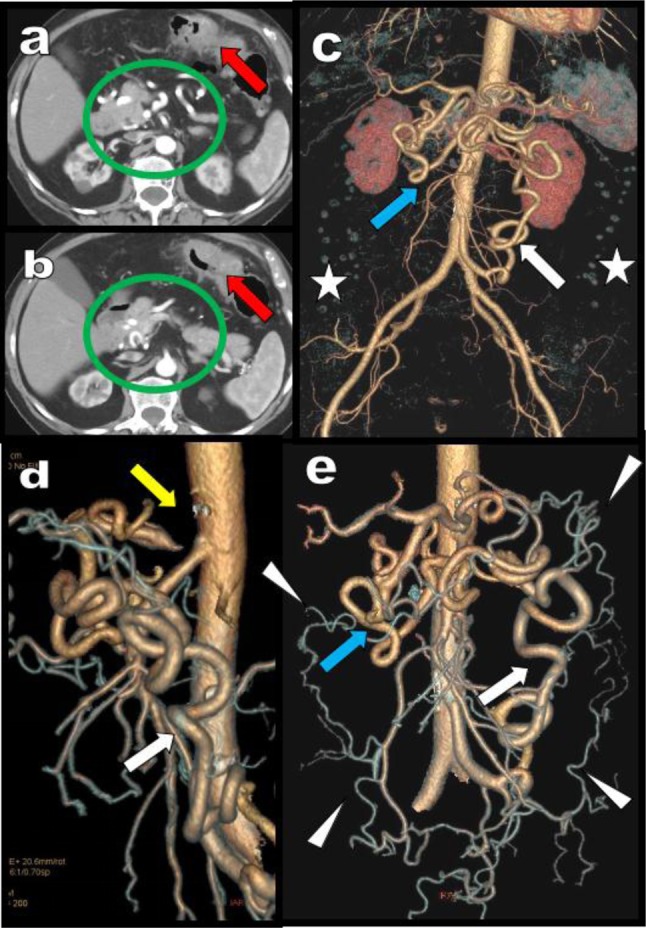
Abdominal MDCTA is performed in this 88-year-old woman for staging of a left transverse colonic adenocarcinoma. Axial views **(a, b)** show the tumour (red arrow) but also unusual development of tortuous arteries in the epigastric area suggesting massive collateralization (green circle). Two groups of huge collaterals are seen on the global VR views **(c, d, e)**. On the right of the aorta huge tortuous hypertrophy of the PDAs (blue arrow) are seen. On the left side of the aorta a huge MeA (white arrow) is visible. This MeA is more centrally located in the mesentery than the more peripheral MAD (white arrowheads). The colonic diverticula are spontaneously also visible and are much more laterally situated in the abdomen than the central MeA (white stars). The cause of collateralization is a complete interruption of the CTK by compression by the MAL (yellow arrow). The absence of stenosis of the emergence of both the SMA and IMA nevertheless allowed safe surgical section through of the MeA during segmental left colonic resection.

During MDCTA the MALS exhibits a characteristic hooked appearance of the focal narrowing of the CTK and the deformation increases during expiration (Figures 1 and 2). This aspect is clearly distinctive from other causes of stenosis such atherosclerosis [9]. Poststenotic dilatation of the compressed CTK is also common as well as the development of collaterals which essentially concern serpiginous hypertrophy of the Gastroduodenal Artery (GDA) and of the cephalic pancreatic arcades (CPAs) (Figures 1 and 3). These antero-inferior and postero-superior CPAs envelop the pancreas head in a circular network [14]. They anastomose the GDA originating from the hepatic artery (HA) with the inferior pancreaticoduodenal artery originating from the SMA usually as its first branch.

It is our opinion that the presence of these collaterals is crucial for the diagnosis of a significant or high degree of compression of the CTK even if the deformation of the CTK is moderate. Indeed, most of the abdominal MDCT are performed in deep inspiration which classically reduces and thus underestimates the compression syndrome. The presence of collaterals helps to diagnose this underestimation. The principle should be: no collateralisation, no MALS except if an additional stenosis on the SMA is present compromising the development of collaterals. Nevertheless, the presence of these collaterals proves that the compression is significant but also demonstrates a good substitution.

As confirmed by several studies the CTCS of MALS is better appreciated during expiration (Figure 2) especially during dynamic duplex and color flow Doppler sonography that are considered by various authors as excellent diagnostic modalities to diagnose significant MALS and to distinguish it from a real atheromatous CTK stenosis in which respiratory variation are absent [7,9,11,12,14].

**Figure 4:**
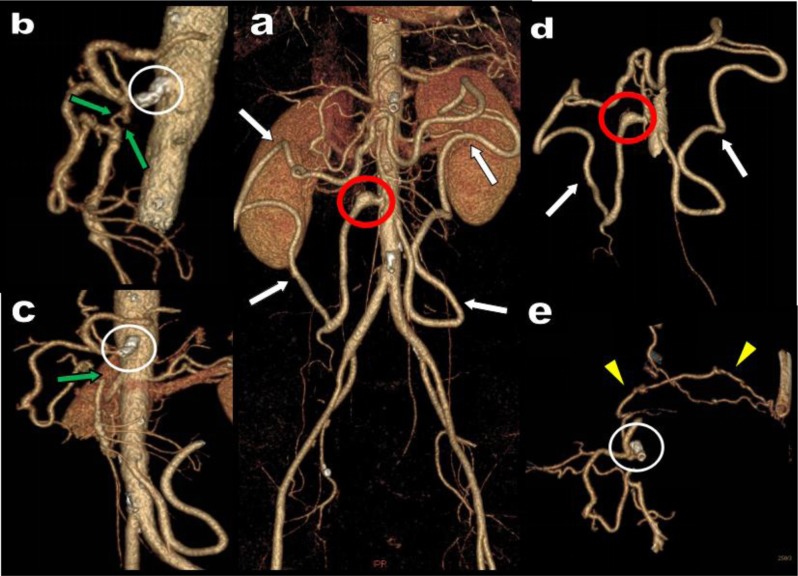
A 55-year-old woman presents with an occlusion of the emergence of the SMA (green arrows). A major stenosis of the CTK due to the MAL had already been treated with a stent (white circle). Nevertheless, this stent appears deformed and twisted suggesting probable restenosis. A very huge and tortuous MeA feed by a hypertrophied IMA has developed all along the mesenteric margin of the large bowel from the sigmoid artery on the left to the ileocolic artery on the right (white arrows). A small aneurysm is found at the end of this arcade just near the anastomose with the distal SMA (red circle). Collateralization of the stenotic CTK also produces by recruitment of the left IPA anastomosed with a left inferior intercostal artery (yellow arrow).

Due to the permanent mechanical extrinsic compression experienced by the CTK in high-grade MALS only a short relief of symptoms followed by early restenosis is classically found after percutaneous angioplasty (PTA) with stenting. The traumatic effects of PTA on the intima and media may weaken the vessel which may become more susceptible to collapse. In addition, stent deployment may be compromised by slippage, mechanical fatigue or crushing secondary to permanent external compression (Figure 4). Therefore, the MALS may be considered as a relative contraindication of PTA or stenting and so compared with the thoracic outlet, the inguinal ligament and the popliteal space [8].

For the same reasons of chronic mechanical compression and major cyclic variation of flow during breathing the potential role of MAL in the development of CTK aneurysm and/or CTK dissection has also been reported (Figure 2) [5,15].

### Double compression of the CTK and SMA by the MAL

**Figure 5:**
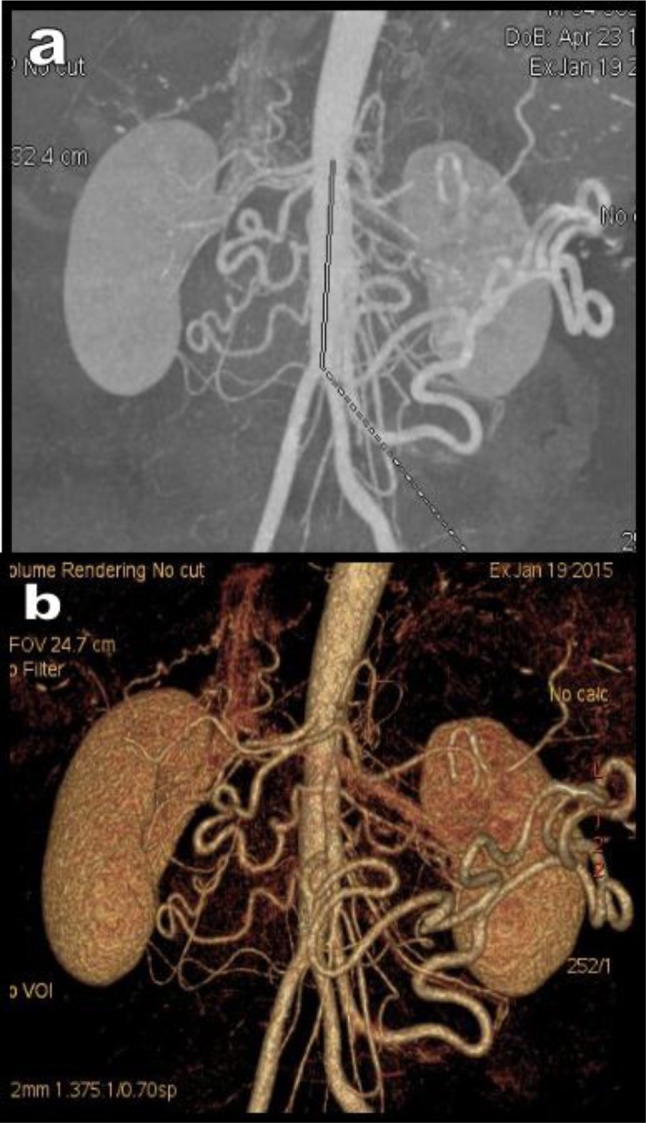
Global MDCTA views (a = MIP view and b = 3D VR view) of the abdominal vessels of a 58-year-old patient show massive diffuse collateralization in the splanchnic area. The aorta is constitutionally short measuring only about 7.5 cm between the emergence of the CTK and the common iliac arteries.

**Figure 6:**
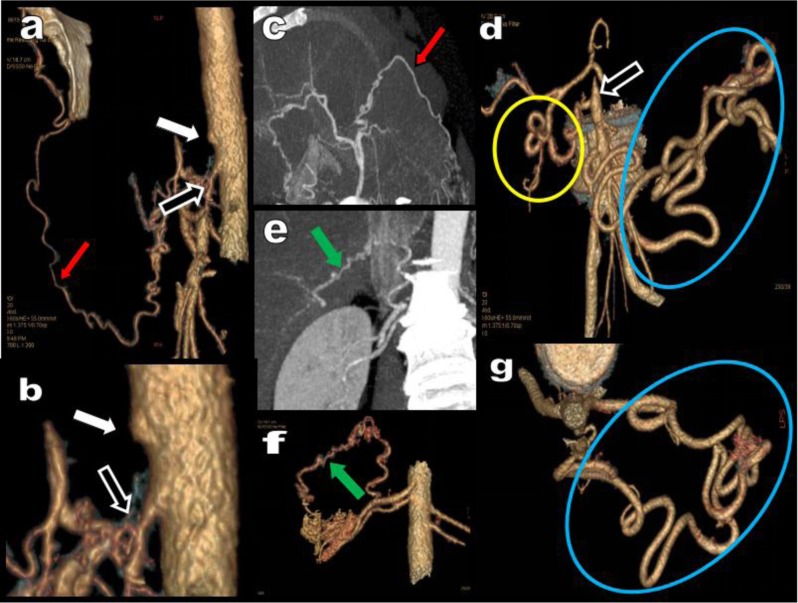
Case of Figure 5 continued. Selective VR views **(a, b, d, f, g)** and MIP views **(c, e)** progressively dissect the complex arterial network. Severe combined occlusion of the CTK (white arrow) and stenosis of the AMS (black arrow) are caused by the MAL of the diaphragm. The HA is secondarily supplied by a long tortuous FA feed by the right ITA (red arrow). Complementary suppleance of the HA also comes from one of the two right RAs (green arrow). Hypertrophy of the PDAs (yellow circle) is also visible and on the left side suppleance of the stenotic AMS is assumed by a vigorous tortuous MeA (blue circle) supplied by the IMA.

**Figure 7:**
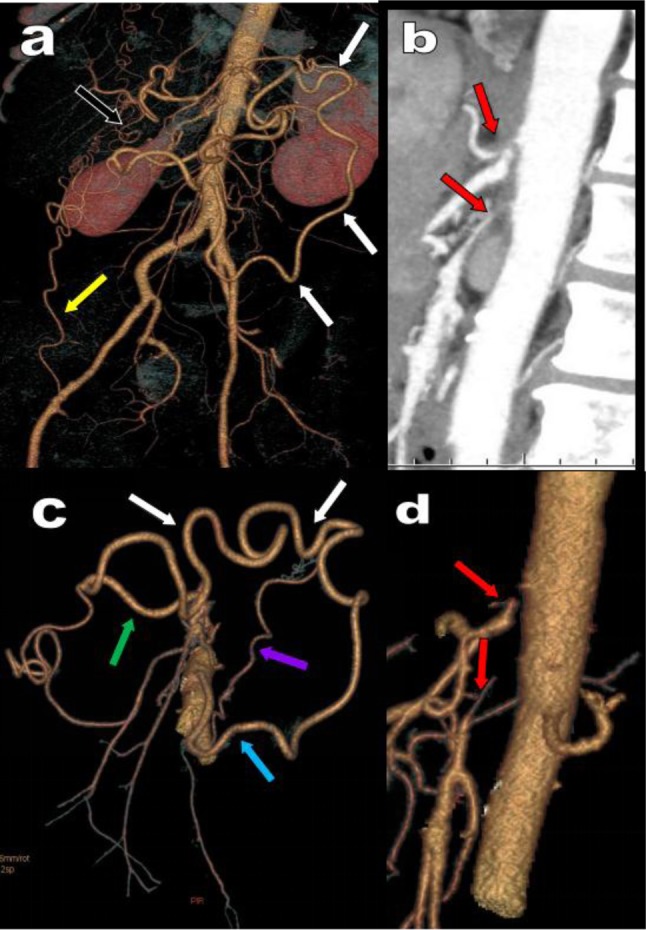
A 54-year-old women presents with a major nearly occlusive stenosis of the CTK and of the AMS (red arrows). Two different networks of suppleance are clearly observed. On the right a small FA (black arrow) feed by the right EA (yellow arrow) supplies the HA. On the left a long tortuous hypertrophy of the MAD (white arrows) supplies the AMS from the level of the left colic artery (blue arrow) to the middle colic artery (green arrow). A thin MeA (or AR) is also present more centrally in the mesentery (purple arrow).

**Figure 8:**
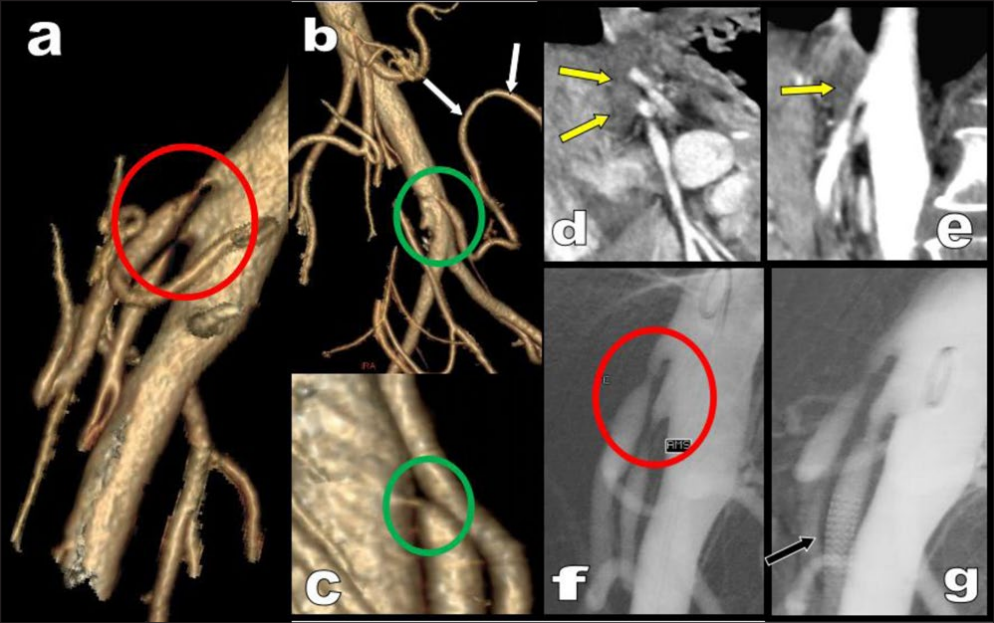
This 58-year-old woman presents with weight loss and chronic abdominal pain suggesting mesenteric claudication or chronic mesenteric ischemia. MDCTA with VR **(a, b, c)** and MIP views **(d, e)** reveals an extremely post-ostial stenosis of both the CTK and SMA (red area) with post stenotic dilatation. The images are typical of a high grade combined compression of the two arteries by a prominent MAL of the diaphragm (yellow arrows). The high-grade stenosis of the CTK is not compensated by any collateralization. The high-grade stenosis of the SMA is supplied by a large MeA (white arrows) but there was nevertheless also a high-grade stenosis of the emergence of the IMA (green circle). Corresponding angiographic images **(f, g)** confirm the high-grade stenosis of both vessels with post stenotic dilatation (red area on f). The stenosis of the SMA is first treated by a stent which unfortunately migrates more distally (black arrow). The interventional procedure is completed by single balloon dilatation of both arteries and a significant improvement of symptoms is obtained.

**Figure 9:**
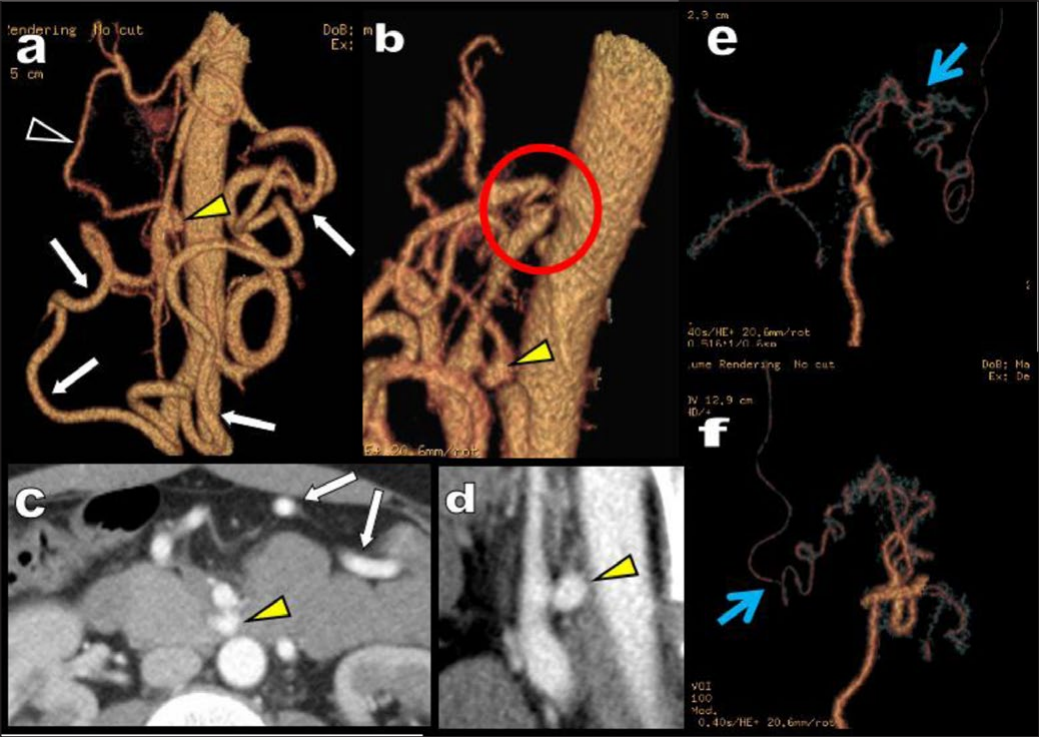
Abdominal MDCTA is performed in this 35-year-old women to exclude RA stenosis or dysplasia in a context of hypertension. RAs arteries are normal (not illustrated) but a very huge tortuous MeA is found (white arrows) on VR **(a)** and axial views **(b)**. The reason is an extreme compression of both the CTK and of the AMS by the MAL (red circle on b). Classical hypertrophy of the PDAs are absent (black arrow) probably because of the combined stenosis of the AMS. A small aneurysm was demonstrated on the MeA just proximally to its anastomose with the AMS (yellow arrowhead) requiring follow up. Selected volume rendering views **(e, f)** also illustrates accessory supply of the stenotic CTK by a small tortuous FA (blue arrow) classically feed by the parietal ITA.

Occasionally, in addition to the CTK the constricting effect of MAL may also manifest on the SMA and rarely on the renal arteries (RAs) [8,9,10]. If the compression on the SMA is important the development of the typical collaterals is compromised and the indirect and more distal substitution by of the IMA may be required (Figures 5, 6, 7, 8 and 9).

Nevertheless, this type of double compression has only been infrequently reported. Only four of the 51 patients with MAL syndrome reported by Reilly had both CTK and SMA compressions [16]. Other isolated cases have also been sporadically reported [12,17,18,19].

### The common celiomesenteric trunk (CCMT)

**Figure 10:**
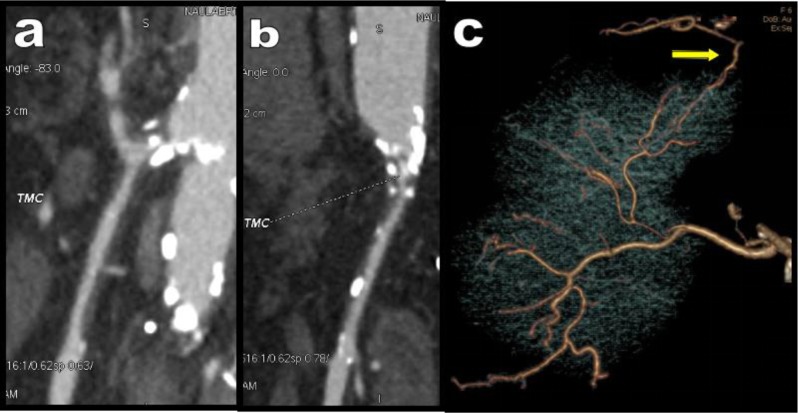
Sagital **(a)** and coronal oblique **(b)** MPR views show a severe atheromatous stenosis of a rare CCMT in this 58-year-old women. Transhepatic anastomose is found between the HA and the right IPA (yellow arrow) as illustrated on this elective VR view **(c)**.

**Figure 11:**
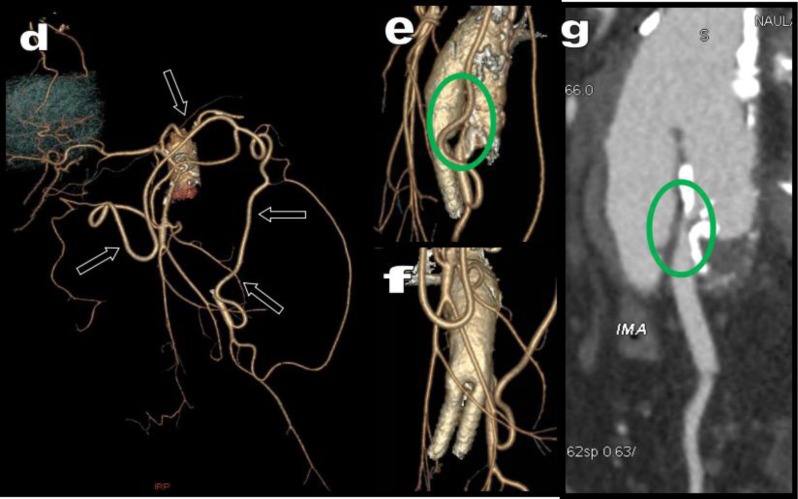
Case of Figure 10 continued **(d, e, f, g)**. Major suppleance of the stenotic CCMT CTMC is assumed by a large MeA (black arrows). Nevertheless, the emergence of the feeding IMA (green circle) also appears critical. This artery emerges just above the distal thombotic segment of the aorta, appears atheromatous with significant stenosis and also appears somewhat wedged between the aorta and the synthetic aorto-iliac bypass.

**Figure 12:**
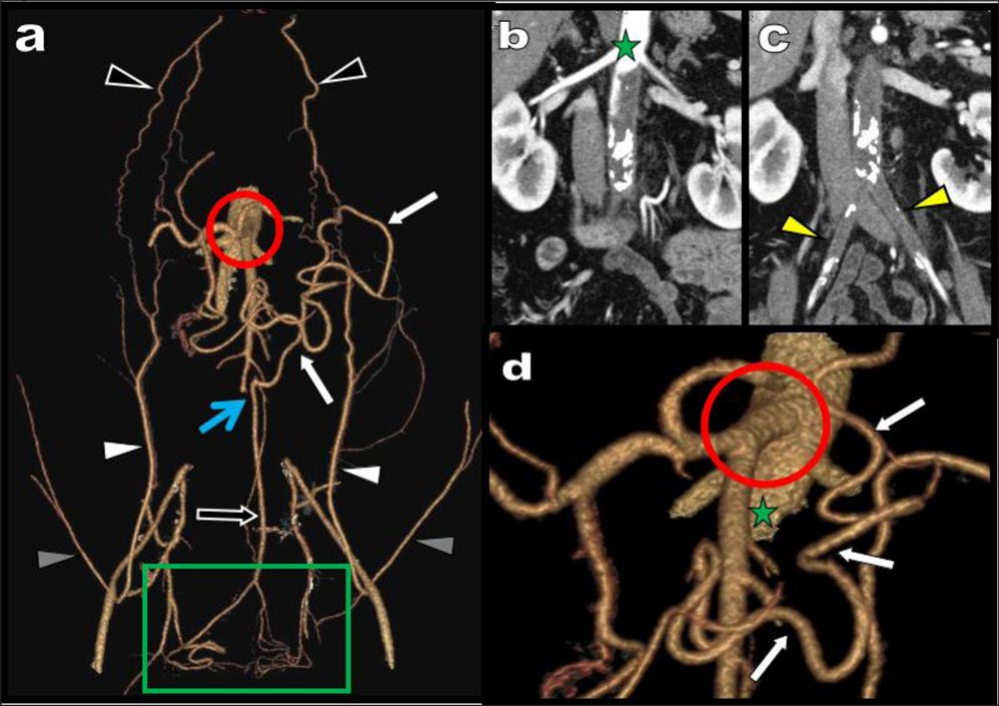
A 55-year-old man presents with LS. A complete thrombotic cut-off of the aortic lumen is found extending from just under the emergence of the RAs (green star) to the distal portion of both common iliac arteries (yellow arrowheads) as shown on these coronal MPR views **(b, c)**. Global VR view **(a)** shows massive collateralizations comprising hypertrophied anastomoses between the EAs (white arrowheads) and the ITAs (black arrowheads) and recruitment of the deep circumflex iliac arteries (grey arrowheads). Massive contribution of the digestives arteries is also present with anastomoses between the hypertrophied IMA (black arrow) and the IIAs via the inferior and medial ReAs (green area on Figure 12a). The emergence of the hypertrophied IMA was nevertheless compromised by the aortic thrombosis (blue arrow). The consequence was the feeding of this hypertrophic IMA by a large MeA (white arrows) developing as a large sinuous anatomose between the IMA and the SMA. Moreover, the SMA had a common aortic emergence with the CA creating a rare but typical CCMT (within the red circle). This variant probably explains the absence of hypertrophied PDAs.

CCMT is a very uncommon variant (Figures 10, 11 and 12) accounting for only 0.25 to 1% of all celiac axis abnormalities and found in 3.4% of a very recent extensive MDCT series of 1,500 patients [13,19,20,21].

A patient with a CCMT is potentially deprived of some of the protective benefits of dual origin vessels with multiple mutually supporting anastomoses. Occlusion or proximal stenosis affecting a common CCMT can have serious ischemic consequences to the intestine because the classical redundancy between the CA and SMA circulation is absent. Moreover, any disorder involving the common CCMT (dissection, thrombosis, emboly, atheromatosis) or an extensive surgery (for example a Wipple’s procedure on the pancreas) may have dramatic consequences on the major abdominal viscera [20,22].

### Secondary aneurysms

Stenosis, occlusion and compression of the CTK by the MAL is known to be one of the main factors for increased collateral circulation and secondarily also to the formation of about 50 to 60% of all pancreatico artery aneurysms (PDAAs) [5,23,24,25].

**Figure 13:**
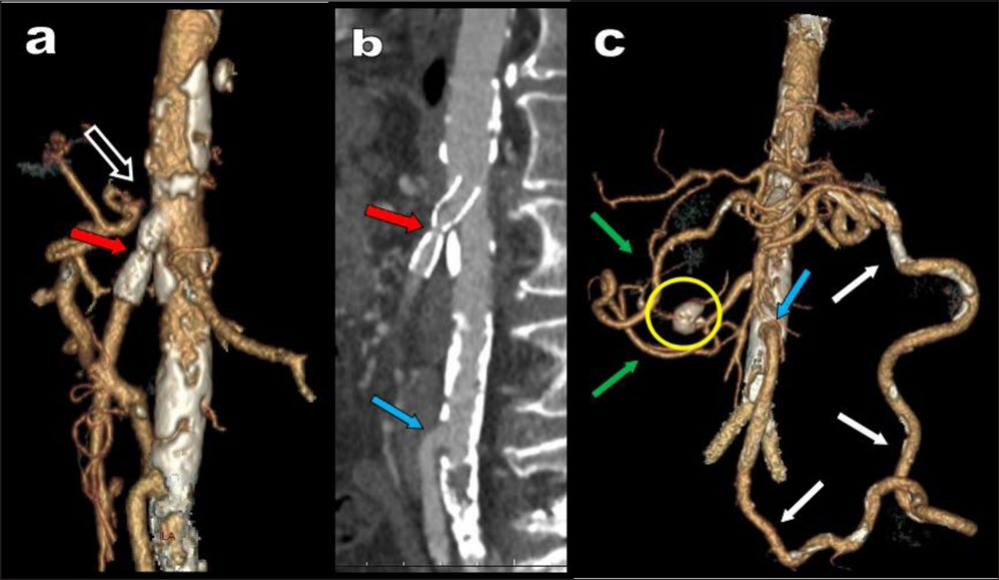
MDCTA is performed in a 74-year-old patient presenting with arteriopathy of the lower limbs. 3D VR views **(a, c)** and sagital MPR **(b)** revealed a high-grade stenosis of the CTK (black arrow) but also a major stenosis of the SMA (red arrow). This stenosis are probably due to a compression by the MAL. The SMA that had already been previously treated by a stent. Nevertheless, this stent was crushed in its central portion with secondary restenosis. The reason is probably the persistence of constant compression by the MAL. Vigorous collateralization is provided by a large diffusely calcified MeA (white arrows). The ostium of the AMI appeared free and large (blue arrows). Suppleance of the stenotic CTK is indirectly obtained through serpiginous hypertrophy of the PDAs (green arrows). A small calcified aneurysm is diagnosed on one of these PDAs (yellow circle).

**Figure 14:**
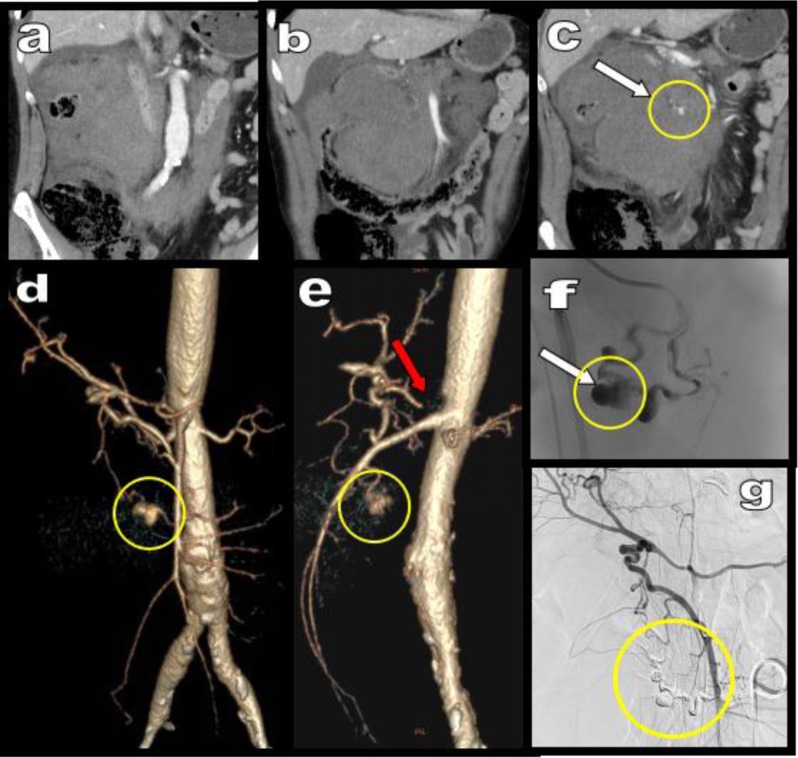
Emergency MDCTA with coronal MPR views **(a, b, c)** illustrates dramatic massive bleeding in the epigastric retroperitoneum of a 57-year-old man. VR views **(d, e)** show occlusion of the CTK by the MAL (red arrow) compensated by hypertrophied PDAs. The active bleeding comes from an aneurysm (white arrow in yellow circle) developed on one of these arcades. Difficult but successful embolization is performed **(f, g)**. (courtesy of F. Deprez, MD, Department of Diagnostic and Interventionnal Radiology, CHU Mont-Godinne, UCL, Belgium).

When compression occurs on the CTK compressed by MAL during expiration the CTK territory becomes abruptly supplied by reverse flow from the SMA through the PDAs causing acute hemodynamic stress in these arteries and promoting aneurysmal formation (Figures 9 and 13) [5,25,26]. These hemodynamic changes may affect the wall shear stress (WSS) of the arteries and a close relationship between a high WSS and the initiation of aneurysm formation has already been demonstrated in animal models [23,24]. PDAAs have also been demonstrated in case of AMS stenosis (Figure 4). Authors [25,26,27] have reported that aneurysm size did not correlate with rupture and suggest that PDAAs should then be treated at the time of diagnosis. The rupture of these aneurysms is a life-threatening emergency (Figure 14).

### Collateral pathways between the SMA and AMI

The IMA is the smallest of the three main mesenteric arteries and supplies the distal transverse, descending and sigmoid colonic segments as well as the rectum [28]. It receives collaterals from the lumbar arteries, the median sacral artery and internal iliac arteries but also has primordial anatomic communications with the SMA. Vascular and abdominal surgeons are aware of this important collateral pathway between the IMA and the SMA. Indeed, inadvertent ligation or section of this important collateral network during aortic or abdominal surgery and especially in the presence of under-diagnosed stenosis of the SMA may have disastrous consequences, especially on the small intestine and left colon [29,30].

The two classical most critical area of watershed of the arterial supply of the left colon are the Griffith’s point in the area of the splenic flexure of the colon were the left branch of the middle colonic artery (branch of the SMA) joins with the ascending branch of the left colonic artery (branch of the IMA) and the Sudeck’s point where collateral communication is found between the last sigmoidal artery and the superior rectal artery, both branches of the IMA.

The collateral pathway between the SMA and the AMI is not always clearly designed. There is a real lack of consensus in the terminology used in the literature causing much confusion. Many denominations are used comprising the arch of Riolan (AR) (considered as a vague historic term to discard) (Figures 16 and 17), the meandering mesenteric artery (MeA) of Moskovitch (actually considered as the more precise term), the central anastomotic artery, the mesomesenteric artery, the middle left colic artery, the anastomosis of Riolan, the meandering artery or the great colic artery of Riolan but also the arch of Treves, the artery of Moskovitch, the anastomosis of Haller and many other names [29,30].

**Figure 15:**
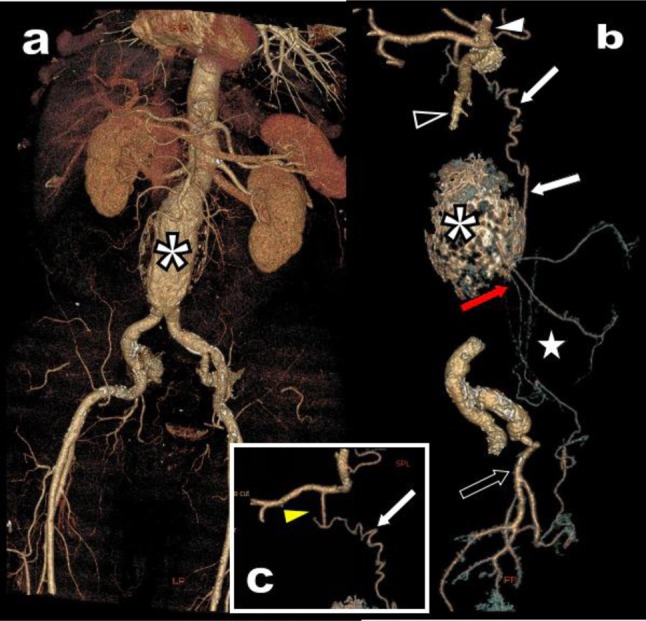
3D VR view **(a)** of the abdominal vessels of a 77-year-old patient presenting with a large infrarenal aortic aneurysm (white asterisk). EVAR is proposed to the patient. 3D selective study of the digestive arterial network **(b, c)** shows CTK (white arrowhead) and SMA (black arrowhead) of very good calibre. Nevertheless, the arterial supply of the left and sigmoid colon appears very precarious (white star). The IMA (red arrow) and its branches have a very thin calibre and will be invariably compromised by the EVAR. Collaterals are poor essentially coming proximally from an atypical serpiginous collateral (white arrows) from the GDA (yellow arrowhead) and distally from rectal collaterals from the left IIA (black arrow). The risk of secondary ischemic colitis or necrosis was substantial but was judged inferior to the risk of spontaneous rupture of the aneurysm. The patient was successfully treated by EVAR.

**Figure 16:**
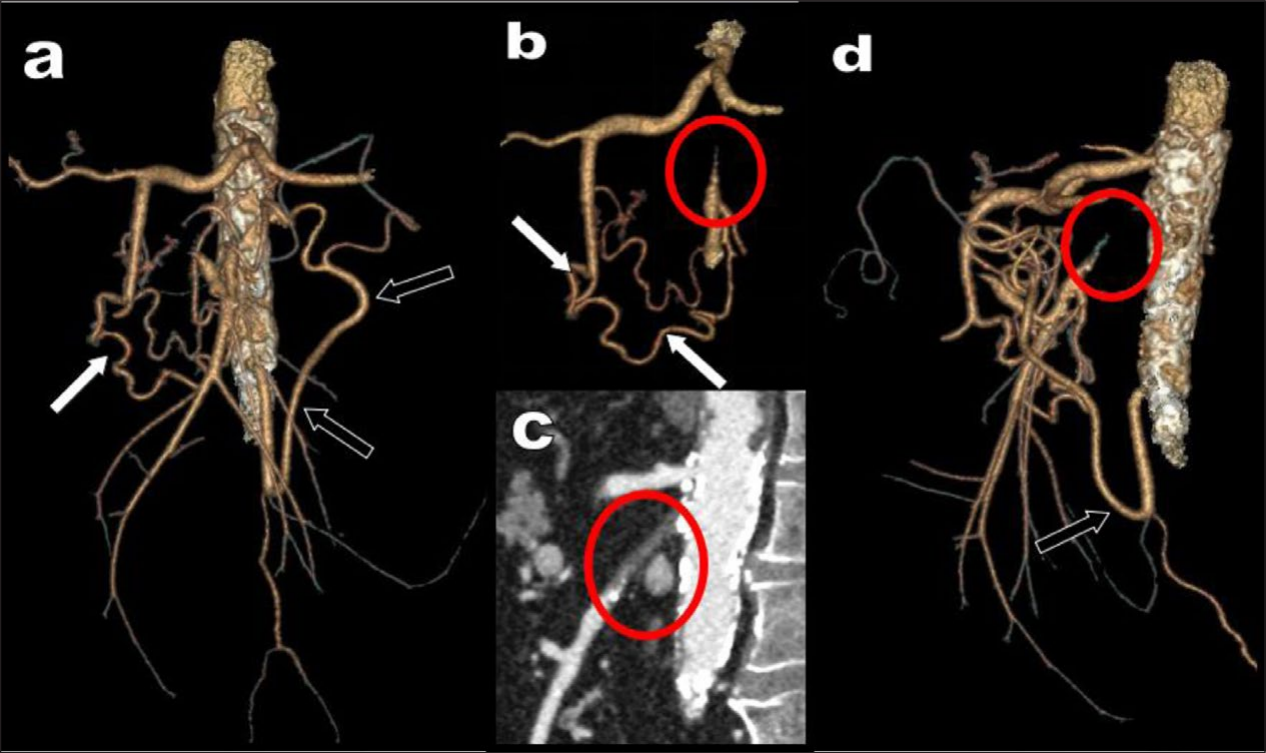
3D views **(a, b, d)** and coronal MPR view **(c)** illustrate classical collateralization in a typical case of isolated high degree atheromatous occlusion of the SMA in a 68-year-old patient (red circle). The stenotic SMA is supplied cranially by serpiginous hypertrophy of the PDAs feed by the CTK (white arrow) and distally by a large MeA feed by the IMA (black arrows).

**Figure 17:**
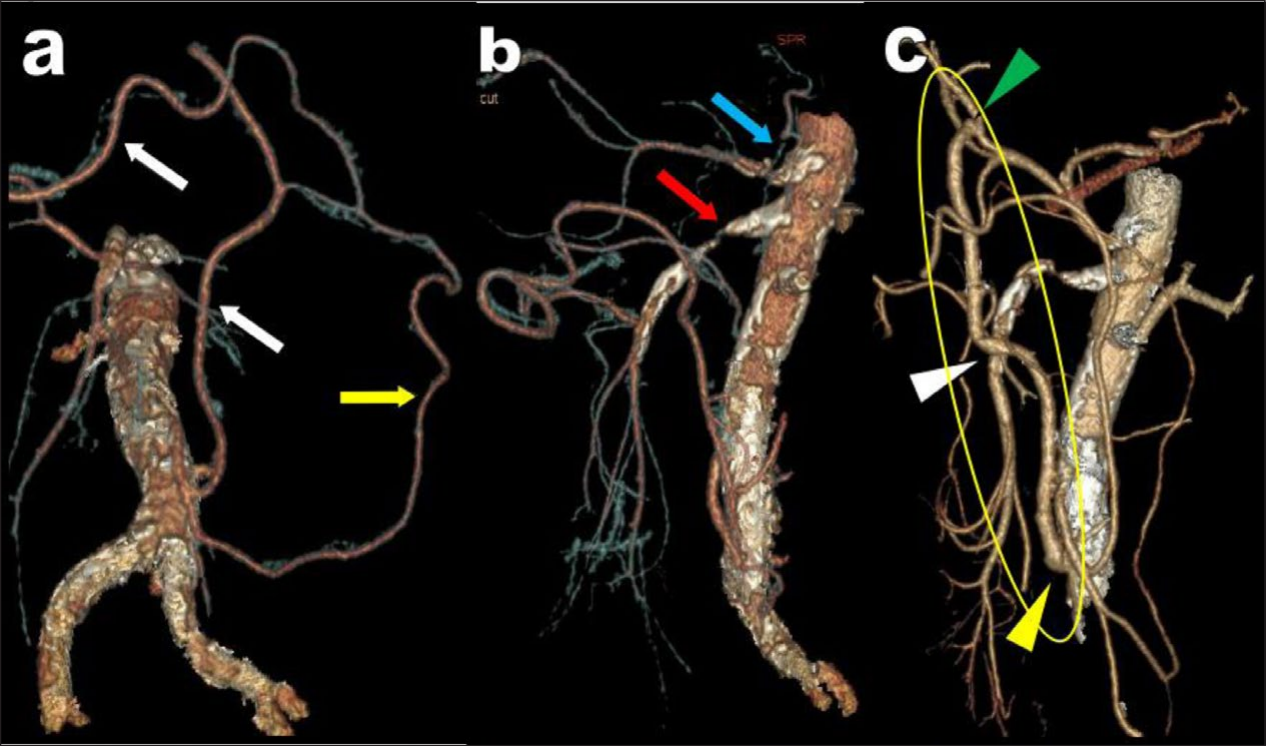
This 53-year-old women presents with symptoms of mesenteric claudication. VR views **(a, b)** of the abdominal vessels reveal an extremely severe atheromatous calcified stenosis of the CTK (blue arrow) and of the SMA (red arrow). A well-structured AR is present (white arrows). It is centrally located and well distinct from the peripheral MAD (yellow arrow). This cartography allows the planning of an optimal surgical treatment **(c)**. A long transmesenteric saphenous-vein bypass graft was performed with an implantation of the lower aorta (yellow arrowhead), a laterolateral anastomose with the SMA (white arrowhead) and a terminal lateral distal anastomose with the HA (green arrowhead).

**Figure 18:**
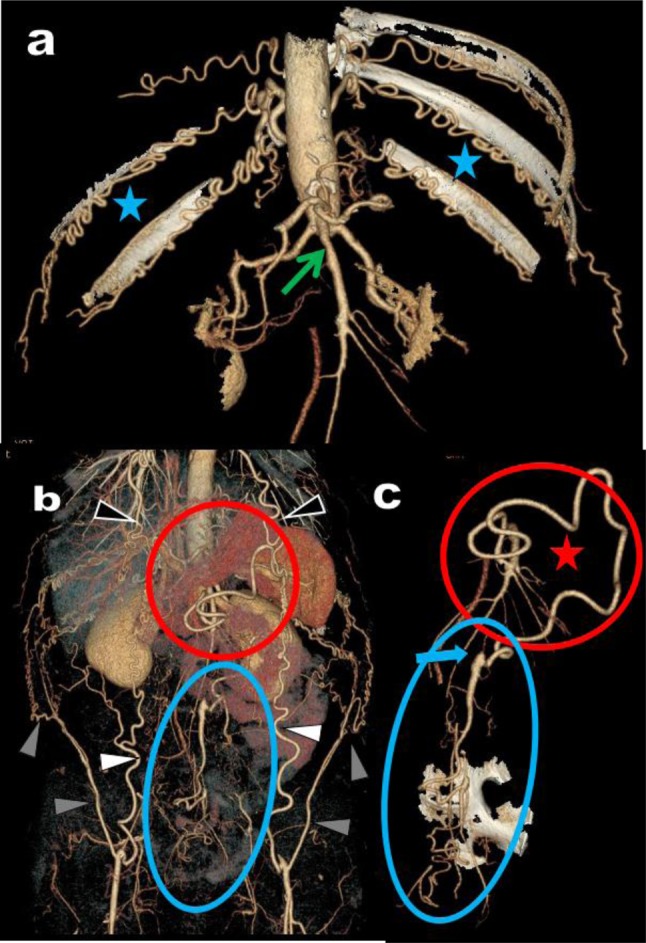
VR views **(a, b)** of the MDCTA of 63-year-old woman presenting with symptoms of LS. A complete thrombotic cut-off of the aorto-iliac system is found extending from just under the emergence of the RAS (green arrow) to the distal portion of both common femoral arteries. Massive collateral arterial network has developed comprising hypertrophied anastomoses between the EAs (white arrowheads) and the ITAs (black arrowheads), massive recruitment of deep circumflex iliac arteries and numerous parietal muscular arteries (grey arrowheads) developing anastomoses with considerable meandering hypertrophy of intercostal arteries (blue stars). An important repercussion on the digestives arteries is also already suspected on the global view (red and blue circles). Selective 3D reconstructions **(c)** confirmed a nearly complete stenosis of the emergence of the IMA (blue arrow) with secondary collateralization by the SMA through a large MeA (red star).

A pragmatic description consists to delineate three concentric different pathways running from the central mesenteric root to its periphery along the colon and comprising:

- Centrally, the inconstant arch of Riolan (AR), joining the middle and left colonic artery and running very close to the inferior mesenteric vein.
- In an intermediate location, the mesentery, the pathway observed in cases of severe stenosis or occlusion of the SMA and known as the Meandering Artery (MeA) (Figures 3, 4, 6, 7, 11, 12, 13 and 18).
- Finally, in the extreme periphery of the mesentery, the marginal arcade of Drummond (MAD), which is classically not tortuous and runs along the left descending colon [28] (Figures 3 and 17).

Many authors consider that the anatomic AR and the MeA of Moskowitz are the same entity, the MeA being the term classically describing the tortuous hypertrophic expansion of the AR in the presence of stenosis or obstruction of the SMA or of the IMA. The expansion of the MeA is greater in presence of stenosis or occlusion of the SMA or in the presence of combined stenosis of SMA and CTK than in isolated stenosis of the IMA because the blood flow load is greater for the SMA than for the IMA [31].

In the presence of a large MeA it is thus recommended to surgeons to abandon or to seriously reconsider their plan for a major resection of the left colon [29]. Secondary necrosis of the right colon and entire small intestine may indeed produce if the flow is reverse in the MeA and necrosis of the sigmoid colon and upper rectum may produce if the flow is antegrade (Figure 3).

### Aorto iliac occlusive disease (AIOD)

AIOD is most frequently a progressive chronic disease resulting of massive deposition of atheromatosis at the level of the aortic bifurcation and on the segment of aorta proximal from this bifurcation. Infrequent causes of AIOD are acute occlusion due to embolus or occlusion related to vasculitis [32]. Nevertheless, many patients remain underdiagnosed because they are asymptomatic as a result of the progressive development of massive rich collateral pathways.

The clinical Leriche’s syndrome (LS) includes the typical triad of symptoms of claudication, impotence and decreased peripheral pulses [33]. In these patients MDCTA cartography is clinically critical for surgical planning and to avoid morbidity of inadvertent surgical injury (Figures 12, 18, 20 and 21) [32].

**Figure 19:**
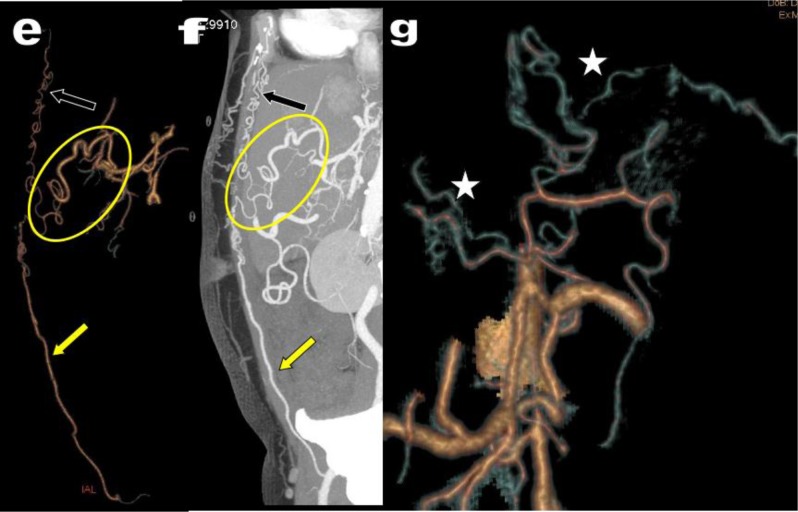
Case of figure 18 continued. Selective 3D **(e)** and 2D **(f)** reconstructions better illustrates the suppleance of the HA by the FA (in the yellow circle) feed by the right EA (yellow arrow) and by the right ITA (black arrow). Complementary suppleance **(g)** of the CA is also assumed by a rich network of oesophageal and phrenic arteries (white stars).

**Figure 20:**
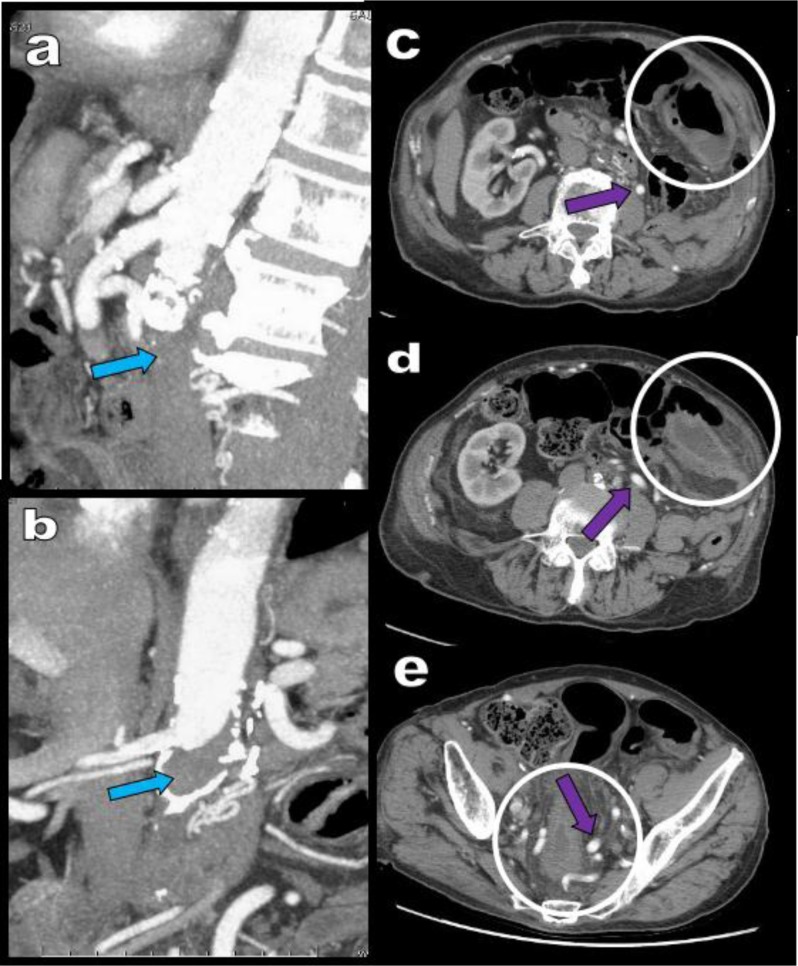
An 84-year-old patient with a known history of metastatic prostate cancer and of LS presents with recurrent episodes of left ischemic colitis confirmed by optical colonoscopy. Sagital **(a)** and coronal **(b)** MPR views show complete thrombosis of the aorta just under the emergence of the RAs (blue arrows). Axial views **(c, d, e)** show typical thickening and hypodensity of the colonic wall (white circle). Huge and massive tortuous arterial collateralization is present along the course of the IMA (purple arrows).

**Figure 21:**
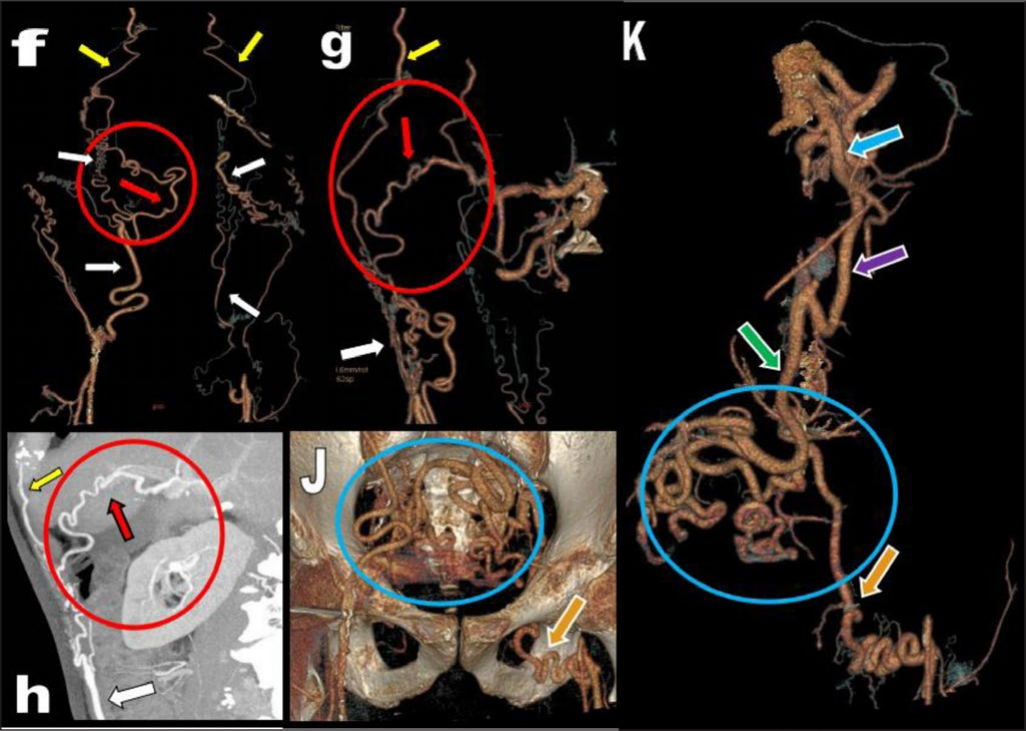
Case of Figure 19 continued. Multiple selective 3D VR reconstructions **(f, g, j, k)** and MIP view **(h)** illustrate huge tortuous collateral arterial network comprising hypertrophied anastomoses between the EAs (white arrowheads) and the ITAs (yellow arrowheads) especially on the right side. Additional collateralisation had developed in the right upper quadrant through recruitment of the HA artery via a large FA (red circle and red arrow). Massive collateralization had also developed in the pelvic area (blue circle) where huge tortuous collaterals coming from the hypertrophied IMA (green arrow) are developing anastomoses with the IIA on the right and with a huge tortuous obturatrice artery (orange arrow) supplying the superficial femoral artery on the left. The emergence of the hypertrophic IMA (green arrow) is occluded by the aortic thrombosis but is supplied by a huge IMT or AoR (purple arrow) coming from the SMA (blue arrow). The episodes of left colonic ischemia are attributed to a steal syndrome from the left leg. Symptoms were successfully healed through a Hartmann procedure comprising segmental colonic resection and left colostomy.

AIOD has different types of collateral pathways which can be classified as visceral-systemic (VS), systemic-systemic (SS) and visceral-visceral (VV) [34].

The VV pathways (deriving from embryologic segments of the ventral aorta) is provided by the CTK, SMA and IMA. This collateral pathway in which the digestives arteries are implicated becomes more prevalent in cases of AIOD extending more proximally along the aorta and thus approaching the level of the emergence of the RAs [32,34].

The SS collateral pathway (deriving from embryologic segments of the dorsal aorta) comprise subcostal, intercostal and lumbar arteries representing the afferent vessels (Figures 12 and 18). They can reconstitute and replace the external iliac arteries (EIAs) through anastomoses with the deep and superficial circumflex arteries or supply the internal iliac arteries (IIAs) through anastomosis with the superior gluteal artery and with the ilio-lumbar artery.

Another SS collateral system is provided by the sacral plexus where the lateral sacral arteries coming from the IIA and the median sacral artery coming from the aorta just above the aortic bifurcation develop collaterals [34].

The internal thoracic artery (ITA) (also called internal mammary artery), and the superior and inferior epigastric arteries (EA) also constitute another SS collateral pathway of primordial importance for the lower limbs (Figures 12 and 18). Inadvertent injury to this parietal vertical system indirectly anastomosing the subclavian arteries with the EIAs – for example during abdominal surgery or because of inadequate recruitment of the ITAs for coronary bypass – may because acute ischemia in the lower limbs [34,35]. Moreover, the inappropriate suppression of this parietal pathway may also intensify the contribution of the visceral arteries (the VS pathway) to supply the lower limbs and initiate chronic mesenteric ischemia or mesenteric claudication.

Finally, the VV pathway is also constituted by a cross pelvic collateral system constituted by communication between the superior, middle and inferior rectal arteries (ReAs) on both sides (Figures 12 and 18).

In each patient presenting with AIOD, the final collateral pattern is individually constituted by a mix of all these above described pathways. It essentially depends of the level of the occlusion: above the IMA, at the level of the IMA or below the IMA. The most proximally the aorta is occluded – from the iliac bifurcation (or under) to just under the level of emergence of the renal arteries – the most important is the recruitment of the digestive arteries and collateralization successively implicates the IMA, the SMA and finally the CTK itself [34]. An optimal cartography of the different possible collateral pathways is thus also necessary to predict the capabilities of the patient to tolerate inadvertent or intentional ligation or embolization of pelvic arteries or pelvic surgery such as left colonic or sigmoid resection (Figure 15).

### The falciform artery (FA)

The FA is a branch of the hepatic artery (HA) that develops anastomoses with the vertical pathway constituted by the EA and the ITA. The FA may thus constitute a VS collateral pathway (in case of Leriche syndrome – LS) or a SV collateral pathway (in case of stenosis of the digestive arteries).

The FA is essentially known by interventional radiologists who perform selective hepatic angiography [13]. They are aware of the potential supraumbilical skin complications which may be produced by inadvertent distribution of chemotherapeutic agents through this artery when they perform transcatheter chemoinfusion or chemoembolization for liver tumours. Otherwise the spontaneous visualization of the HFA is very uncommon in current abdominal CT practice [13].

To our knowledge, there are no studies reporting the prevalence of the FA detection during dynamic CT studies in healthy patients. Nevertheless, our opinion, based on our personal experience with 64-row multidetector CT is that this prevalence remains extremely low [13].

In a previous report, we reported two cases in which it was likely that the FA was visualized because it was enlarged by a compensatory phenomenon related to the critical state of digestive arteries of the patients. One had a CCMT and the other had severe compression of the CTK and of the SMA by the MAL [13]. Additional cases are illustrated in this pictorial review and were found in cases of LS (Figure 21) or in cases of compression of the CTK by the MAL (Figures 6 and 19).

### Other unusual collateral pathways

Left and right inferior phrenic arteries (IPAs) are other rare arteries being able of collateralization with the HA or with the CTK (Figure 19). As for the HFA, these arteries are also known by interventional radiologist as potential extrahepatic collateral arteries [36]. They may supply hepatic adenocarcinoma or act as collaterals in patient presenting with stenosis of the CTK (Figures 4 and 10). Unusual extrahepatic collaterals may also concern accessory RAs (Figure 6).

**Figure 22:**
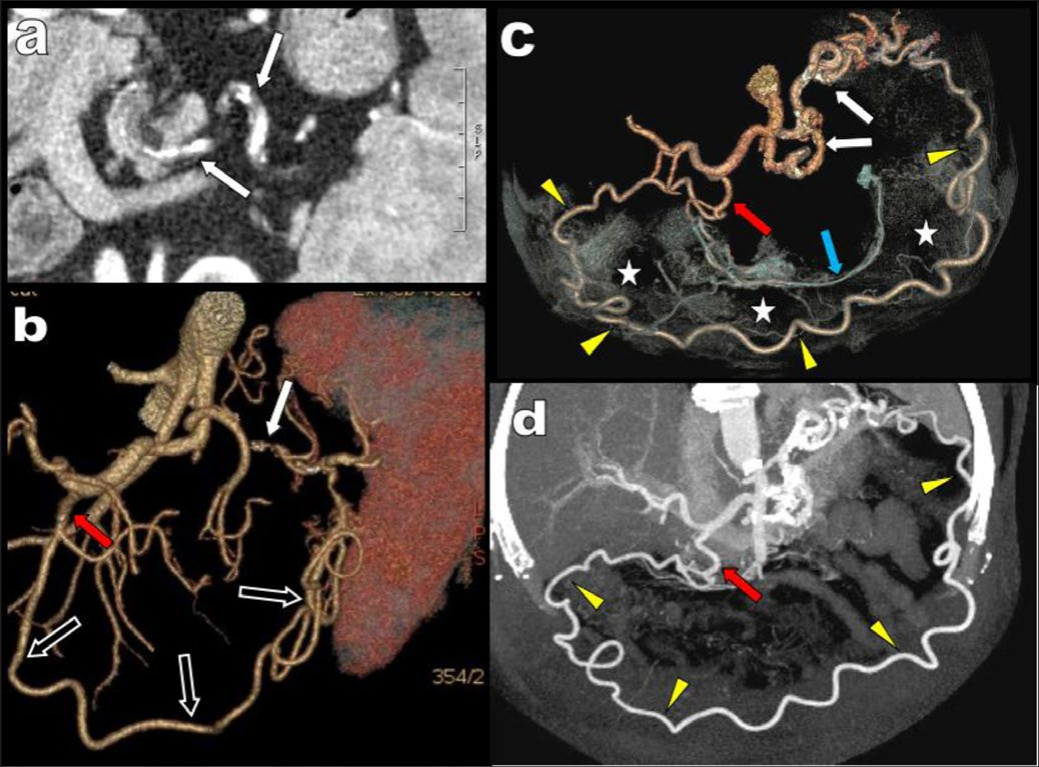
A major calcified atheromatous stenosis (white arrow) is found on the distal SA of this 67-year-old man **(a, b)**. The arterial supply of the spleen is preserved through an important collateralization by the hypertrophied main GEA (black arrows) a branch of the GDA (red arrow). In this 58-year-old patient **(c, d)** an extremely rare collateralization of a stenosed atheromatous SA (white arrow) is constituted by a huge meandering hypertrophy of the omental arterial arcade of Barkow (yellow arrowhead). This arcade is also feed by the GDA (red arrow).

In our experience, we also recently found two cases of atheromatous stenosis of the splenic artery (SA) fortuitously diagnosed through the presence of unusual collateralisation. The first case was collateralized by a tortuous gastroepiploic artery (GEA) a situation which has only exceptionally been described in rare cases of absence or occlusion of the SA [37] (Figure 22). The other case was supplied by an enormous meandering hypertrophy of the arcus epiploica magnus of Barkow. To our knowledge this last type of collateralisation has never been described before (Figure 22).

## Conclusion

Through this extensive pictorial review, we have illustrated a large diversity of complex abdominal situations implicating the digestive arteries and/or the systemic abdominal arteries, the two arterial systems being frequently interconnected. These situations are not uncommon in clinical practise. We confirm and demonstrate that multidetector computed tomographic angiography (MDCTA) can be very effective not only to diagnose a single arterial stenosis or compression but also to dissect combined and/or complex associations of multiple stenosis and/or compressions of several arteries. MDCTA also appears uncompetitive unavoidable to map sometimes very complex networks of collateralization.

MDCTA is confirmed being the gold standard for the diagnostic evaluation of abdominal and/or mesenteric arterial diseases. It represents a primordial advance to plan the safety of the digestive vascularisation before many major abdominal surgical procedures and to plan revascularization of the mesenteric arterial system itself.

## List of Abbreviations

AIOD = AortoIliac Occlusive Disease

AR = Arcade of Riolan

CA = Conventional Angiography

CACS = Celiac Artery Compression Syndrome

CCMT = Common CelioMesenteric Trunk

CPAs = Cephalic Pancreatic Arcades

CTK = Celiac Trunk

DIA = Diaphragmatic Artery

EA = Epigastric Artery

EIAS = External Iliac Arteries

EVAR = EndoVascular Aneurysm Repair

GDA = Gastroduodenal Artery

FA = Falciform Artery

GEA = GastroEpiploic Artery

HA = Hepatic Artery

IIAs = Internal Iliac Arteries

IMA = Inferior Mesenteric Artery

IMT = Intermesenteric Trunk

IPA = Inferior Phrenic Artery

ITA = Internal Thoracic (or mammary) Artery

LS = Leriche Syndrome

MAD = Marginal Arcade of Drummond

MAL = Median Arcuate Ligament

MALS = Median Aarcuate Ligament Syndrome

MDCTA = Multidetector Computed Tomography Angiography

MeA = Meandering Artery

PDAA = Pancreatico Duodenal Artery Aneurysm

PDAs = Pancreatico Duodenal Arteries

PTA = Percutaneous Angioplasty

ReAs = Rectal Arteries

RAs = Renal Arteries

SA = Splenic Artery

SMA = Superior Mesenteric Artery

SS = Systemic-Systemic

VR = Volume Rendering

VSC = Visceral-Systemic

VVC = Visceral-Visceral

WSS = Wall Shear Stress
